# Revealing Microvascular Involvement in Pediatric Localized Scleroderma Through Nailfold Capillaroscopy

**DOI:** 10.3390/children12091245

**Published:** 2025-09-17

**Authors:** Sema Nur Taşkın, Şeyda Doğantan, Esra Esen, Sümeyra Özdemir Çiçek, Ayşenur Paç Kısaarslan, Muammer Hakan Poyrazoğlu

**Affiliations:** 1 Division of Pediatric Rheumatology, Department of Pediatrics, Faculty of Medicine, Erciyes University, 38039 Kayseri, Türkiye; 2 Department of Pediatrics, Division of Pediatric Rheumatology, Eskişehir City Hospital, Eskişehir 26080, Türkiye

**Keywords:** juvenile localized scleroderma, nailfold capillaroscopy, microvascular alterations, mLoSSI/LoSDI, 6 min walk test, pediatric rheumatology

## Abstract

**Highlights:**

**What are the main findings?**

- Capillary tortuosity, crossing, dilatation, and neoangiogenesis were more frequently observed in jLoS patients.
- Patients showed significantly higher capillary density, length, arterial limb widths, apical loop width, and disorganization scores.
- No avascular areas or giant capillaries were detected in either group.

**What is the implication of the main finding?**

- Nailfold capillaroscopy may serve as a valuable non-invasive tool for early vascular assessment in jLoS.

**Abstract:**

**Background/Objectives**: Juvenile localized scleroderma (jLoS) is a chronic inflammatory disorder with skin and subcutaneous tissue involvement. Microvascular alterations are thought to contribute to its pathogenesis. This study aimed to investigate microvascular alterations in children with jLoS using nailfold capillaroscopy (NFC) and to compare the capillaroscopic findings between patients and healthy controls. **Methods:** A total of 13 children diagnosed with jLoS and 16 age- and sex-matched healthy controls were enrolled. Capillaroscopic assessment included capillary density, tortuosity, dilatation, disorganization, branching, and neoangiogenesis. Dilated and giant capillaries, hemorrhages, avascular areas, and capillary loss were evaluated. The Microangiopathy Evaluation Score (MES) was used to semi-quantitatively assess capillary loss, disorganization, and ramifications. Disease activity and damage were evaluated using the modified Localized Scleroderma Skin Severity Index (mLoSSI) and the Localized Scleroderma Damage Index (LoSDI), respectively. Functional status was measured via the 6 min walk test (6MWT). **Results:** Plaque morphea was the most common subtype (61.5%), and antinuclear antibody (ANA) positivity was present in 53.8% of patients. Compared to controls, jLoS patients exhibited significantly more frequent capillaroscopic abnormalities, including increased tortuosity, crossing, dilatation, and neoangiogenesis (*p* < 0.05). Capillary density, length, arterial limb width, apical loop width, and disorganization scores were significantly higher, while intercapillary distance was lower in jLoS patients (*p* < 0.05). No avascular areas or giant capillaries were observed. MESs were similar between groups. **Conclusions:** NFC revealed significant microvascular alterations in jLoS patients, supporting its utility as a non-invasive tool for early vascular assessment in localized scleroderma.

## 1. Introduction

Localized scleroderma (LoS), also known as morphea, is a rare chronic inflammatory disorder of the connective tissue that can present with diverse clinical manifestations in both pediatric and adult populations. It is primarily characterized by inflammation and fibrosis of the skin and subcutaneous tissues, and in some cases may extend to deeper structures such as fascia, muscle, bone, and even the central nervous system [1,2].

Nailfold Capillaroscopy is a validated, non-invasive, and reproducible imaging technique used to assess microvascular architecture, particularly in connective tissue diseases. It is considered a key diagnostic and monitoring tool in systemic sclerosis, and its application is increasingly being explored in other rheumatologic and dermatologic conditions with suspected microangiopathy [3,4,5].

Although localized scleroderma and systemic sclerosis (SSc) differ in severity and progression, it has been hypothesized that they share a common pathogenic vascular component. Recent studies support this hypothesis by demonstrating that NFC abnormalities—such as capillary dilatation, architectural disorganization, and neoangiogenesis—can also be observed in patients with localized scleroderma, particularly in the pediatric population. However, prior pediatric LoS studies have reported heterogeneous NFC findings. Early reviews emphasized the absence of major SSc-type abnormalities in LoS, whereas more recent multicenter pediatric work has described quantitative alterations—most commonly reductions in capillary density—in subsets of children with rheumatic diseases. In parallel, reports integrating clinical features and serology suggested that, in morphea/LoS, NFC tends to capture early or limited microvascular involvement rather than overt scleroderma-pattern damage [2,5]. Against this mixed background—and the specific inconsistency surrounding capillary density—we sought to characterize pediatric LoS using standardized image acquisition and semi-quantitative scoring, and to compare these features with age- and sex-matched healthy controls. By explicitly contrasting qualitative morphology and quantitative metrics, our study aimed to clarify whether pediatric LoS predominantly exhibits early, non-SSc patterns on NFC.

The 6-minute walk test is widely used to assess functional capacity and cardiopulmonary endurance in patients with (SSc) and juvenile systemic sclerosis (jSSc). However, its application in LoS is limited and not well-established in the literature [6]. Therefore, we employed the 6MWT to evaluate functional capacity in pediatric LoS patients.

Building on this background, we evaluated disease activity and cumulative tissue damage in pediatric LoS using the Modified Localized Scleroderma Severity Index (mLoSSI) and the Localized Scleroderma Damage Index (LoSDI) as part of the Localized Scleroderma Assessment Tool (LoSCAT). In addition, patients’ NFC findings were compared with those of age-matched healthy controls, and their exercise capacity and functional endurance were assessed through the 6MWT.

## 2. Materials and Methods

This cross-sectional prospective study was conducted at the Department of Pediatric Rheumatology, Erciyes University Faculty of Medicine, and included the evaluation of NFC findings in children diagnosed with LoS. The study protocol was approved by the Erciyes University Clinical Research Ethics Committee (Decision No. 2021/804; Date: 8 December 2021). All procedures were carried out in accordance with the principles outlined in the Declaration of Helsinki. Written informed consent was obtained from the parents or legal guardians of all participating children.

Patients: Patients were classified according to the most widely used clinical subtypes: circumscribed morphea, linear scleroderma, generalized morphea, pansclerotic morphea, and the mixed subtype, in which two or more of the aforementioned subtypes coexist [7]. Between August and December 2021, NFC examinations were performed in 13 pediatric patients diagnosed with jLoS. Patients with any additional systemic or rheumatologic disorders were excluded from participation. Patients included in the study were carefully evaluated to ensure that they had not experienced any infections at the time of enrollment or in the recent past.

Definitions: The LoSCAT is a validated clinical instrument designed to evaluate both disease activity and damage in patients with localized scleroderma. It comprises two components—mLoSSI for assessing activity, and the LoSDI for evaluating permanent changes—across 18 predefined anatomical sites [8].

The 6MWT is a simple, standardized, and widely used submaximal exercise test that measures the distance an individual can walk on a flat, hard surface in six minutes. It reflects integrated cardiopulmonary and musculoskeletal function and is commonly used to assess functional capacity and endurance in both adult and pediatric populations with chronic conditions. The 6MWT has been widely applied in patients with systemic sclerosis, where it serves as a practical tool to evaluate exercise tolerance and monitor disease progression or treatment response, particularly in cases with cardiopulmonary involvement [9]. In studies conducted on healthy children and adolescents, 6MWT distances have been shown to vary by age and sex; however, the overall mean distance is 618 ± 79 m [10]. As this study was conducted during the COVID-19 pandemic, pulmonary function tests (PFT) and diffusion capacity of the lung for carbon monoxide (DLCO) were not performed. To minimize hospital stay and reduce the potential risk of viral transmission, these procedures were omitted in both patients and healthy controls. Instead, the six-minute walk test (6MWT), a practical and safe method for evaluating functional capacity and cardiopulmonary endurance, was employed, while standard reference values from the literature were considered for healthy children.

Nailfold Capillaroscopy: After resting for at least 20 min at room temperature (20–24 °C), capillaroscopic assessments were conducted using a video microscope (MEDL4N Dino-Lite Pro Capillary Scope, Dino-Lite Europe, Almere, The Netherlands). All fingers, excluding the thumbs, were examined, and all image acquisitions and final evaluations were performed by a single pediatric rheumatologist with three years of experience in nailfold capillaroscopy.

The evaluation encompassed morphological abnormalities such as capillary loss, branching, and dilatation; hemorrhagic areas; avascular zones; neovascular alterations assessed via a neoangiogenesis score; capillary disorganization; and capillary density per millimeter [11,12]. Capillaries with an internal diameter of 25–50 μm were classified as dilated, while those with a diameter of ≥50 μm were defined as giant capillaries [12]. Morphological criteria were determined based on previously established definitions. The evaluated parameters included the total number of capillaries across the nailfold width, the mean nailfold capillary density (NFCD) per millimeter, capillary size, and the number of dilated, giant, branching, and tortuous capillaries. In line with previous definitions, capillary tortuosity, crossing, and mild dilatation (<50 μm) were categorized as ‘minor abnormalities’ that may also occur in healthy subjects [11,13]. The MES for each patient was calculated as described by Sulli et al. [13]. This semi-quantitative scale assesses capillary loss, disorganization of the microvascular architecture, and capillary ramifications, each scored from 0 to 3. The total MES is obtained by summing the individual component scores, resulting in a range from 0 to 9.

Study Groups: The study group consisted of 13 children diagnosed with juvenile localized scleroderma (jLoS group). A total of 16 healthy children without any known medical conditions who agreed to participate were selected as the control group (control group). The hemogram parameters, blood pressure measurements, height, and weight of the children in the control group were all within age-appropriate reference ranges.

Variables: Data were collected and recorded on demographic characteristics (age, sex), clinical features (age at diagnosis, physical findings, and medications), blood pressure measurements, and laboratory parameters. Capillaroscopic and clinical data were collected cross-sectionally during outpatient follow-up visits after the initiation of the study.

Statistical Analysis: Descriptive statistics for continuous variables were expressed as mean ± standard deviation, median, minimum, and maximum values, while categorical variables were presented as counts and percentages. The Shapiro–Wilk test was used to assess the normality of data distribution.

Comparisons of continuous variables between the patient and control groups were performed using the Mann–Whitney U test. For comparisons of nominal variables across groups (in cross-tabulations), the Chi-square test or Fisher’s exact test was used as appropriate. All statistical analyses were conducted using IBM SPSS Statistics version 20 (IBM Corp., Chicago, IL, USA). A *p*-value of <0.05 was considered statistically significant. Age and gender distributions were comparable between patients and controls, and no significant differences were observed in univariate analyses. Regression analysis was not applied because of the limited sample size and the lack of variability in capillary morphology within the patient group, which would have precluded meaningful modeling.

## 3. Results

A total of 29 children, comprising 13 patients with juvenile localized scleroderma and 16 age- and sex-matched healthy controls, were enrolled in the study. The patient group was predominantly female (76.9%), and the mean age was 12.9 years. No statistically significant differences were found between the groups in terms of age or sex distribution (*p* > 0.05). All patients exhibited white blood cell (WBC) counts and erythrocyte sedimentation rate (ESR) values within normal reference ranges, indicating the absence of active systemic inflammation. Among the disease subtypes, 8 patients (61.5%) had plaque morphea, 3 (23.0%) had linear morphea, 1 (7.6%) had the mixed subtype (linear + plaque), and 1 (7.6%) had the generalized subtype. Regarding treatment regimens, 4 patients were receiving methotrexate (MTX) in combination with topical therapy, 3 were treated with mycophenolate mofetil and topical agents, and 6 patients were managed with topical therapy alone. ANA positivity was detected in 7 patients. The patients’ age, follow-up duration, blood pressure (BP), hemoglobin (Hb), platelet count (PLT), 6MWT performance, mLoSSI, and LoSDI values are presented in Table 1. Spearman correlation analysis demonstrated a significant negative correlation between 6MWT and mLoSSI (r = −0.56, *p* = 0.048), indicating that higher disease activity was associated with reduced functional capacity. A negative correlation was also observed between 6MWT and LoSDI (r = −0.51, *p* = 0.074), although this did not reach statistical significance, likely due to the limited sample size.

However, a statistically significant difference was observed in capillary morphology between the patient and control groups (*p* < 0.01) (Table 2).

**Table 2:** Comparison of Age, Sex, and Nailfold Capillaroscopic Morphological Findings Between Children with Localized Scleroderma and Healthy Controls. Data represent cross-sectional findings obtained during outpatient follow-up visits at the time of study enrollment.

	Patients (*n* = 13)		Controls (*n* = 16)		*p*-Value
	Mean ± SD/Median (Min–Max)		Mean ± SD/Median (Min–Max)		
Age	12.92 ± 3.04 12 (9–17)		12.81 ± 3.44 12.5 (8–17)		0.914 ^a^
	***n***	**%**	***n***	**%**	
Gender					
Female	10	76.9	12	75.0	1.000 ^b^
Male	3	23.1	4	25.0	
Capillary morphology					
Normal	0	0	8	50.0	0.003 ^b^
Minor Abnormality	13	100	8	50.0	

^a^: Mann–Whitney U test. ^b^: Chi-square test/Fisher’s exact test. In the patient group, capillary density, capillary length, arterial limb width, apical loop width, capillary width, and disorganization scores were found to be significantly higher compared to the control group. In contrast, the intercapillary distance was significantly lower in the patient group than in the control group (Table 3).

**Table 3:** Comparison of Nailfold Capillaroscopic Parameters Including Density, Length, Width, Intercapillary Distance, and Disorganization Between Patients with Localized Scleroderma and Healthy Controls. Data represent cross-sectional findings obtained during outpatient follow-up visits at the time of study enrollment.

	Patients (n = 13)	Controls (n = 16)	
Parameter	Mean ± SD/Median (Min–Max)	Mean ± SD/Median (Min–Max)	*p*-Value
Density (/mm)	11.31 ± 1.70 11 (8–14)	8.56 ± 1.1155 8.5 (7–11)	**<0.001 ^a^**
Capillary Length (µm)	422.00 ± 119.92 380 (260–623)	232.44 ± 40.58 220 (177–313)	**<0.001 ^a^**
Arterial Limb Width (µm)	11.46 ± 1.45 11 (10–15)	10.06 ± 1.23 10 (8–13)	**0.009 ^a^**
Venous Limb Width (µm)	14.46 ± 1.80 14 (12–17)	13.50 ± 1.21 13.5 (12–16)	0.170 ^a^
Apical Loop Width (µm)	16.00 ± 1.87 15 (13–18)	14.50 ± 1.31 15 (12–17)	**0.050 ^a^**
Capillary Width (µm)	39.92 ± 3.57 41 (32–45)	37.31 ± 3.32 38 (30–42)	**0.040 ^a^**
Intercapillary Distance (µm)	89.38 ± 12.09 90 (75–120)	104.19 ± 17.51 102 (80–136)	**0.009 ^a^**
Disorganization Score	0.25 ± 0.15 0.37 (0–0.38)	0 (0–0)	**<0.001 ^a^**

^a^: Mann–Whitney U test. Bold formatting highlights statistically significant values (*p* < 0.05).

In the patient group, the frequencies of tortuosity, crossing, dilated capillaries, capillary meandering, and neoangiogenesis were significantly higher compared to the control group (Table 4). No giant capillaries or avascular areas were detected in either the patient or control groups.

There were no statistically significant differences between the patient and control groups in terms of neoangiogenesis score, microhemorrhage score, and MES (Microangiopathy Evaluation Score (Table 5).

## 4. Discussion

The present investigation evaluated NFC findings and functional capacity in jLoS compared with healthy controls. Regarding quantitative parameters, patients with jLoS demonstrated significantly higher values of capillary density, capillary length, arterial limb width, apical loop diameter, overall capillary width, and disorganization scores, while the intercapillary distance was markedly reduced. Qualitative morphological assessment revealed that capillary tortuosity and crossing were universally present in patients and differed significantly from controls. Dilated capillaries were observed exclusively in the jLoS group, whereas giant capillaries and avascular areas were absent in both groups. In addition, capillary meandering and neoangiogenesis were detected only in patients, while bushy capillaries and microhemorrhages occurred infrequently and were restricted to the patient group. Functional assessment with the 6MWT demonstrated significantly reduced capacity in children with jLoS compared to reference values, indicating that microvascular involvement may also contribute to impaired physical performance. Collectively, these findings highlight that jLoS is not merely a skin-limited condition but is associated with measurable microvascular alterations and functional impairment, even at early disease stages.

The clinical and laboratory characteristics of the jLoS patients evaluated in this study are consistent with profiles reported in the existing literature. The female predominance observed in our cohort (76.9%) is consistent with previous literature, including a 2023 review (4:1 ratio) and a 2025 Canadian study of 6063 LoS cases (74.4% female, ~3:1 ratio) [1,14].

**Figure 1:**
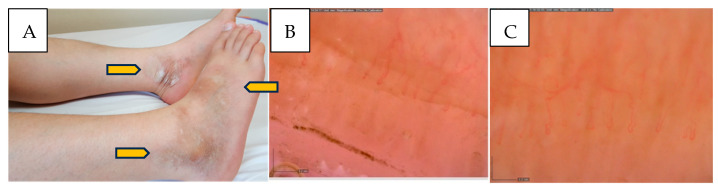
(**A**–**C**). Clinical and capillaroscopic findings in a patient with juvenile localized scleroderma.

In this study, plaque morphea was identified as the most common subtype of jLoS, accounting for 61.5% of cases (Figure 1). Although this finding differs from several previous international studies, it remains consistent with the variability reported in the literature. In a large multicenter international study conducted by Zulian et al. (2006) involving 750 pediatric patients, linear morphea was the most prevalent subtype (65.9%), while plaque morphea accounted for 35.6% of cases [2]. Similarly, Egeli et al. (2025) reported linear morphea as the most frequent subtype in their tertiary pediatric cohort of 101 patients in the United States [15]. However, the distribution of LoS subtypes appears to vary substantially depending on geographical, ethnic, and methodological factors. For instance, a retrospective cohort study conducted in Turkey in 2022 found that plaque morphea was the most common subtype among 39 children [16]. In this context, the predominance of plaque morphea in our study may reflect regional and demographic characteristics of the patient population. Additionally, the generalized and mixed subtypes were detected in 7.6% of our patients, which aligns with previous reports indicating that these variants are rare in pediatric populations [2].

Regarding treatment, four patients received a combination of methotrexate (MTX) and topical therapy, three were treated with mycophenolate mofetil (MMF) in conjunction with topical agents, and six received topical therapy alone. MTX remains the most frequently used first-line systemic agent for jLoS, and its efficacy has been demonstrated in randomized controlled trials. A retrospective study by Singhal et al. (2023) reported that MMF is a safe and effective therapeutic option for jLoS patients who are resistant or intolerant to MTX [17].

ANA positivity is a commonly reported serologic finding in jLoS. In the multicenter study conducted by Zulian et al., ANA positivity was found in approximately 42% of pediatric patients [2]. In our cohort, ANA positivity was detected in 53.8% of patients, which is consistent with prior data. Moreover, before NFC evaluation, systemic parameters (blood pressure, C-reactive protein (CRP), hemoglobin, and platelet count) were assessed, and all were within normal limits. This indicates that the observed NFC alterations were unlikely to be secondary to systemic inflammation, thereby supporting the interpretation that these changes reflect localized vascular involvement. Although ANA positivity was observed in 53.8% of our cohort and nearly half of the patients received systemic therapy, subgroup analyses were not feasible due to the limited sample size. Therefore, potential influences of ANA serostatus or treatment exposure on microvascular findings could not be statistically evaluated, which represents a limitation of our study.

Figure 1A: Shows the clinical appearance of plaque morphea lesions in a 10-year-old girl, involving the lateral malleolar region and dorsum of the right foot, as well as the medial malleolar region of the left foot. The lesions are characterized by hyperpigmentation, induration, and mild atrophy.

Figure 1B,C: In both 200× magnification images, the capillary loops exhibit a tortuous and irregular course, while no giant capillaries, hemorrhages, neoangiogenesis, or significant capillary dropout are observed. The preservation of overall capillary density suggests a “non-SSc pattern”; however, the observed morphological alterations indicate early microvascular involvement associated with localized scleroderma.

Beyond demographic and laboratory features, functional capacity and vascular involvement were also explored in our cohort. There is a limited number of studies in the literature addressing the use of the 6MWT specifically in jLoS. In a study conducted by Köker et al., the mean walking distance was reported as 480 m in 25 patients with juvenile systemic sclerosis. In the same study, the mean distance among 30 healthy controls was 553 m [6]. In studies involving healthy children and adolescents, 6MWT distances have been shown to vary by age and sex; however, the overall mean walking distance is approximately 618 m [10]. In our study, the mean walking distance in jLoS patients was 499 m. These findings suggest that although jLoS patients demonstrate some degree of reduced functional performance compared to healthy peers, the impairment is not as severe as that observed in systemic diseases such as jSSc. Therefore, our study provides an important contribution to the literature regarding the applicability of 6MWT in children with jLoS. The functional capacities of the patients were evaluated using the 6MWT, while disease activity and tissue damage were assessed using mLoSSI and LoSDI, respectively. This integrative assessment strategy allowed for a more holistic evaluation of jLoS, linking microvascular findings with functional capacity, disease activity, and cumulative tissue damage.

In this study, significant differences in capillary morphology were observed when comparing NFC findings between jLoS patients and healthy controls. Although no statistically significant differences were found between the groups in terms of age and sex, capillary morphology showed a statistically significant distinction. In their 2013 review titled “Nailfold Capillaroscopy in Pediatrics,” Francesca Ingegnoli and Ariane L. Herrick emphasized that major capillary abnormalities had not been identified in patients with LoS, while also highlighting the limited number of studies addressing this topic [18]. In a multicenter study conducted by Melsens et al., which included 21 jLoS patients, capillary density was found to be significantly lower in LoS patients compared to healthy controls. However, no statistically significant differences were observed between the jLoS group and controls in terms of capillary dilatation or abnormal shapes. These findings suggest that jLoS may involve distinct microvascular alterations, although these changes may be less pronounced than those seen in systemic SSc and other connective tissue diseases [5]. In a study conducted by Doğantan et al., decreased capillary density and increased disorganization were reported in children with juvenile dermatomyositis [19]. NFC is widely used for the early diagnosis and monitoring of microvascular changes in other connective tissue diseases such as SSc [20]. Mostmans et al. emphasized the diagnostic and prognostic value of NFC in patients with morphea and SSc by integrating clinical skin findings and serological markers. Their findings support the utility of NFC in evaluating microvascular alterations even in limited disease forms such as LoS [21]. Similarly, Ornowska et al. demonstrated that NFC was effective in detecting microvascular abnormalities in patients with mixed connective tissue disease (MCTD), and that these changes may be associated with internal organ involvement [22]. Di Pino et al. also underscored the utility of NFC as a valuable tool for the early diagnosis of connective tissue diseases beyond the scleroderma spectrum. These studies collectively highlight the relevance of NFC in identifying vascular changes in limited forms like LoS [23]. Our findings expand on this evidence, suggesting that jLoS is associated with measurable but relatively subtle microvascular alterations.

In our study, jLoS exhibited significantly higher values of capillary density, capillary length, arterial limb width, apical loop diameter, overall capillary width, and disorganization scores compared to healthy controls. In contrast, intercapillary distance was found to be significantly reduced in the patient group. Taken together, these findings point toward early or non–SSc–type vascular involvement, rather than advanced remodeling. Although the sample size was small, the observed differences corresponded to large effect sizes (e.g., density difference ≈ 2.75/mm), which supports their potential clinical relevance.

In the patient group, the frequency of tortuous capillaries, capillary crossing, dilated capillaries, meandering loops, and neoangiogenesis was significantly higher compared to the control group. In contrast, no cases of giant capillaries or avascular areas were observed in either group. The absence of these hallmark SSc features (e.g., giant capillaries, avascular areas) indicates that the microvascular alterations observed in jLoS are relatively limited and should not be over-interpreted as equivalent to systemic disease–related patterns. Instead, our findings support the notion that jLoS involves microvascular changes, but these remain less advanced than the severe microangiopathy typically seen in SSc. Consistently, studies by Melsens et al. (2022) and Mostmans et al. (2021) also reported that although capillary morphology is altered in LoS, these changes are not as widespread or severe as those observed in SSc [5,21]. In this context, NFC appears to play an important role in detecting early microvascular changes in LoS, and qualitative parameters such as capillary disorganization may potentially correlate with disease activity.

The classification of “scleroderma-type” capillaroscopic changes, as currently defined by Cutolo et al., is widely accepted and consists of three distinct phases: “early,” “active,” and “late.” The early phase is characterized by the presence of a few giant capillaries (megacapillaries) and a limited number of microhemorrhages, with preservation of capillary distribution and density. In the active phase, the number of megacapillaries and microhemorrhages increases, accompanied by moderate capillary loss and architectural disorganization. The late phase is marked by pronounced capillary derangement, widespread avascular areas, and the presence of neoangiogenic or bushy capillary loops [24].

In our study, no statistically significant differences were found between jLoS patients and healthy controls in terms of neoangiogenesis scores, microhemorrhage scores, or Microangiopathy Evaluation Score (MES). This absence of hallmark SSc features (e.g., giant capillaries, avascular areas) suggests that the jLoS microvascular profile is distinct and limited, and should not be over-interpreted as equivalent to systemic disease patterns. This finding suggests that although LoS affects the microvascular architecture, it is not characterized by the overt neovascularization or capillary hemorrhage patterns typically observed in more aggressive systemic diseases such as SSc. The similarity of these parameters between the patient and control groups further supports the notion that LoS, while altering capillary morphology, may not result in advanced microvascular remodeling or endothelial damage [24,25,26]. Additionally, the comparable MES values between groups suggest a relatively limited burden of overall capillary damage in LoS patients. These findings indicate that vascular involvement in LoS is likely more localized and less extensive than in systemic connective tissue diseases. Consequently, NFC may serve as a useful tool for the early differentiation of localized diseases such as LoS, particularly in distinguishing them from more systemic vasculopathies [27].

In recent years, advanced imaging modalities have expanded the scope of microvascular assessment beyond traditional NFC. Jasionyte et al. (2023) successfully utilized Superb Microvascular Imaging (SMI) to characterize microvascular patterns in patients with systemic sclerosis. This technique offers higher-resolution visualization of vascular structures and may provide a more sensitive and detailed evaluation of vascular changes in patients with LoS. Future incorporation of such imaging modalities could complement NFC, enabling more refined characterization of early vascular alterations in jLoS and contributing to better disease stratification [28].

### 4.1. Limitations

This study has several limitations that should be acknowledged. The sample size was relatively small, which may reduce the statistical power and generalizability of the findings. Moreover, the distribution of localized scleroderma subtypes was not uniform, with the majority of patients presenting with plaque morphea. Although plaque morphea is considered the mildest clinical form of jLoS, capillaroscopic abnormalities were still detectable, highlighting the potential of NFC even in early or less severe disease presentations. Furthermore, regression analysis could not be performed to explore potential confounding effects of age and gender on capillary morphology because of the limited sample size and the lack of variability in morphological findings within the patient group, which would have precluded meaningful modeling. All capillaroscopic images were obtained and evaluated by a single experienced examiner, and inter-rater reliability was not assessed. This methodological limitation may reduce the reproducibility of our findings. Additionally, although each capillaroscopic parameter was analyzed independently as a distinct hypothesis, the evaluation of multiple parameters without formal correction (e.g., Holm–Bonferroni or FDR) may increase the risk of type I error. This potential bias should be considered when interpreting our findings. In addition, although ANA positivity was observed in more than half of the patients and nearly half were under systemic therapy, subgroup or correlation analyses could not be performed due to the small sample size. Therefore, potential influences of ANA serostatus or treatment exposure on microvascular findings could not be statistically evaluated.

The cross-sectional design precludes any assessment of temporal changes in microvascular parameters. Longitudinal follow-up studies are necessary to determine whether capillaroscopic alterations progress over time or respond to treatment in jLoS. Furthermore, while NFC provides valuable structural information about the microvasculature, it does not offer functional data such as tissue perfusion or endothelial function. The integration of advanced imaging modalities, such as Superb Microvascular Imaging (SMI), may help overcome this limitation in future research.

This study was conducted during the COVID-19 pandemic, which limited the performance of pulmonary function tests (PFT) and diffusion capacity of the lung for carbon monoxide (DLCO). Consequently, functional capacity and cardiopulmonary endurance were assessed exclusively by the 6MWT, while reference values from the literature were used for healthy controls. Although the 6MWT is a validated and practical tool, the absence of direct pulmonary function measurements may have restricted the comprehensive evaluation of cardiopulmonary status.

Finally, although efforts were made to exclude patients with systemic involvement or active infection, subclinical factors that could influence microvascular morphology may still have been present.

### 4.2. Clinical Implications and Future Research

The findings of this study suggest that in patients with jLoS, the microvascular structure may be affected not only morphologically but also functionally and clinically. NFC, as a non-invasive technique, emerges as a valuable tool for detecting early microvascular alterations and monitoring disease progression in LoS. In particular, qualitative capillary parameters such as disorganization may be associated with disease activity. Therefore, it is recommended that NFC be incorporated into routine assessment protocols in pediatric dermatology and rheumatology clinics.

Future longitudinal studies should investigate the relationship between capillaroscopic findings and clinical subtypes, risk of systemic involvement, and therapeutic response. Moreover, the application of next-generation imaging modalities such as SMI in LoS may allow for more sensitive and detailed evaluation of microvascular pathology. Functional tests—such as the 6MWT should be applied in larger cohorts to reveal potential subclinical musculoskeletal involvement. Future research should aim to increase sample sizes, compare different LoS subtypes, evaluate treatment responses through capillaroscopic monitoring, and develop standardized assessment protocols.

## 5. Conclusions

This study highlights that jLoS involves not only cutaneous but also significant subclinical microvascular alterations. Despite the absence of systemic inflammation, distinct capillaroscopic abnormalities were identified, supporting the role of microangiopathy in the pathogenesis of jLoS. The integration of NFC, clinical indices (mLoSSI and LoSDI), and the 6MWT enabled a comprehensive evaluation of disease activity, cumulative tissue damage, and functional capacity. Marked differences in capillary morphology—particularly increased tortuosity, crossing, and disorganization—are compatible with early or non-SSc microvascular involvement rather than advanced remodeling

These findings reinforce the utility of NFC as a non-invasive, dynamic tool for early detection and longitudinal monitoring in LoS. Incorporating NFC into routine clinical assessments may enhance diagnostic precision and facilitate more targeted therapeutic strategies. Future longitudinal and multicenter studies using advanced imaging modalities are warranted to further elucidate the vascular landscape of localized scleroderma.

## Figures and Tables

**Table 1:** Demographic, Clinical, and Functional Characteristics of Patients with Localized Scleroderma.

Patient ID	Sex	Involvement Type	Age (Years)	FU (Years)	BP (mmHg)	Hb (g/dL)	PLT (×10^3^/µL)	6MWT (Meters)	mLoSSI	LoSDI
Patient 1	F	plaque morphea	9	3	100/60	13	278	456	9	10
Patient 2	M	Mixed subtype (linear + plaque morphea	9	3	100/60	13.9	380	456	10	32
Patient 3	F	plaque morphea	10	6	90/60	12.6	336	450	6	14
Patient 4	F	plaque morphea	10	2	100/60	12.1	461	500	0	4
Patient 5	F	plaque morphea	11	4	100/60	14.3	241	600	0	8
Patient 6	F	plaque morphea	12	3	100/60	13.8	342	515	0	8
Patient 7	F	plaque morphea	12	4	100/60	13.8	339	480	0	16
Patient 8	F	linear morphea	14	7	100/60	14.3	318	550	15	22
Patient 9	M	linear morphea	15	3	110/70	13.7	310	500	0	15
Patient 10	F	linear morphea (en coub de sabre)	16	5	100/60	13.9	367	500	5	23
Patient 11	F	plaque morphea	16	4	110/70	13	300	516	0	8
Patient 12	M	plaque morphea	17	5	110/70	14.7	218	554	0	10
Patient 13	F	Generalize subtype	17	10	100/60	15.1	189	420	43	60

FU: Follow-up; F: Female; M: Male; BP: Blood Pressure; Hb: Hemoglobin; PLT: Platelet count; 6MWT: 6-Minute Walk Test—a measure of functional exercise capacity; mLoSSI: Modified Localized Scleroderma Severity Index—used to assess disease activity; LoSDI: Localized Scleroderma Damage Index—used to evaluate permanent tissue damage in localized scleroderma.

**Table 4:** Comparison of Morphological Capillaroscopic Findings. Data represent cross-sectional findings obtained during outpatient follow-up visits at the time of study enrollment.

Finding	Patients (n = 13) n (%)	Controls (n = 16) n (%)	*p*-Value
Increased Tortuosity	13 (100.0%)	8 (50.0%)	**0.003 ^a^**
Capillary Tortuosity	None: 0 (0.0%) <50%: 1 (7.7%) >50%: 12 (92.3%)	None: 8 (50.0%) <50%: 8 (50.0%) >50%: 0 (0.0%)	**<0.001 ^a^**
Increased Crossing	13 (100.0%)	2 (12.5%)	**<0.001 ^a^**
Capillary Crossing	None: 0 (0.0%) <50%: 3 (23.1%) >50%: 10 (76.9%)	None: 14 (87.5%) <50%: 2 (12.5%) >50%: 0 (0.0%)	**<0.001 ^a^**
Dilated Capillaries	9 (69.2%)	0 (0.0%)	**<0.001 ^a^**
Giant capillary	0(0.0%)	0(0.0%)	0
Avascular area	0(0.0%)	0(0.0%)	0
Capillary Meandering	4 (30.8%)	0 (0.0%)	**0.030 ^a^**
Bushy Capillaries	3 (23.1%)	0 (0.0%)	0.078 ^a^
Neoangiogenesis	4 (30.8%)	0 (0.0%)	**0.030 ^a^**
Microhemorrhages	1 (7.7%)	0 (0.0%)	0.078 ^a^

^a^: Chi-square test/Fisher’s exact test. Bold formatting highlights statistically significant values (*p* < 0.05).

**Table 5:** Nailfold Capillaroscopy-Based Scoring of Morphological Abnormalities in Pediatric Localized Scleroderma and Controls.

	Patients (*n* = 13)	Controls (*n* = 16)	
Parameter	Mean ± SD/Median (Min–Max)	Mean ± SD/Median (Min–Max)	*p*-Value
Tortuosity Score	0.77 ± 0.24 0.87 (0.25–1.00)	0.20 ± 0.22 0.12 (0–0.50)	**<0.001 ** ^a^
Crossing Score	0.66 ± 0.30 0.50 (0.25–1.0)	0.03 ± 0.08 0 (0–0.25)	**<0.001 ** ^a^
Dilated Capillary Score	0.31 ± 0.26 0.25 (0–0.75)	0 (0–0)	**0.001 ** ^a^
Meandering Score	0.25 ± 0.41 0 (0–1.13)	0 (0–0)	0.170 ^a^
Bushy Score	0.06 ± 0.13 0 (0–0.38)	0 (0–0)	0.308 ^a^
Neoangiogenesis Score	0.18 ± 0.33 0 (0–1.0)	0 (0–0)	0.170 ^a^
Microhemorrhage Score	0.19 ± 0.06 0 (0–0.25)	0 (0–0)	0.746 ^a^
MES	0.07 ± 0.27 0 (0–1.0)	0 (0–0)	0.746 ^a^

^a^: Mann–Whitney U test. Bold formatting highlights statistically significant values (*p* < 0.05).

## Data Availability

The datasets generated and/or analyzed during the current study are available from the corresponding author (S.N.T.) upon reasonable request. The data are not publicly available due to patient privacy and ethical restrictions.

## Abbreviations

6MWT	Six-Minute Walk Test
ANA	Antinuclear Antibody
DLCO	Diffusion Capacity of the Lung for Carbon Monoxide
jLoS	Juvenile Localized Scleroderma
LoS	Localized Scleroderma
LoSCAT	Localized Scleroderma Cutaneous Assessment Tool
LoSDI	Localized Scleroderma Damage Index
mLoSSI	Modified Localized Scleroderma Severity Index
NFC	Nailfold Capillaroscopy
PFT	Pulmonary Function Test
SMI	Superb Microvascular Imaging
SSc	Systemic Sclerosis
